# Draft Genome Sequence of the Type Strain Lysobacter capsici VKM B-2533

**DOI:** 10.1128/MRA.01194-20

**Published:** 2021-01-21

**Authors:** Sergey V. Tarlachkov, Irina V. Kudryakova, Alexey S. Afoshin, Maria N. Tutukina, Elena A. Leontyevskaya, Natalia V. Vasilyeva

**Affiliations:** a G. K. Skryabin Institute of Biochemistry and Physiology of Microorganisms, Russian Academy of Sciences, FRC PSCBR RAS, Moscow Region, Pushchino, Russia; b Center of Life Sciences, Skolkovo Institute of Science and Technology, Moscow, Russia; c Institute of Cell Biophysics, Russian Academy of Sciences, FRC PSCBR RAS, Moscow Region, Pushchino, Russia; University of Maryland School of Medicine

## Abstract

Lysobacter capsici VKM B-2533^T^ is a promising strain for isolation of new lytic agents. Here, we report a draft genome sequence of this strain, consisting of 131 scaffolds with a total length of 6,196,943 bp. The results obtained will aid in the discovery and study of biologically active compounds important for biomedicine.

## Abstract (precis)

Lysobacter capsici VKM B-2533^T^ is a promising strain for isolation of new lytic agents. Here, we report a draft genome sequence of this strain, consisting of 131 scaffolds with a total length of 6,196,943 bp. The results obtained will enable us to find and study biologically active compounds important for biomedicine.

## ANNOUNCEMENT

Bacteria of the genus *Lysobacter* are unique producers of various lytic agents (bacteriolytic enzymes, antibiotics, and peptides) (1–7). In this regard, *Lysobacter* spp. are of great interest for biomedicine and agriculture. Lysobacter capsici VKM B-2533^T^ (=KCTC 22007^T^ = DSM 19286^T^) was isolated from the rhizosphere of pepper at the Gyeongsang National University (Jinju, South Korea) in 2008 (8). This strain was shown to have potent antifungal and antibacterial effects (7–9). An antifungal effect against Phytophthora infestans was observed in volatile organic compounds found in the strain (10). Bacteriolytic enzymes were also purified, among them β-lytic protease, which demonstrated potent antistaphylococcal activity (7). Here, we report a draft genome sequence of *L. capsici* VKM B-2533^T^.

*L. capsici* VKM B-2533^T^ was obtained from the All-Russian Collection of Microorganisms (VKM). The strain was cultivated in KSP medium (11) at 29°С with aeration for 18 h. DNA was extracted using the QIAamp DNA minikit (catalog no. 51304) according to the manufacturer’s instructions. Sequencing was performed by the Genomics Core Facility at the Skolkovo Institute of Science and Technology. A short-read library was prepared with the NEBNext Ultra II kit (New England Biolabs, USA) according to the manufacturer’s recommendations. The library was sequenced on the NextSeq 500 platform (Illumina, USA).

Default parameters were used for all software unless otherwise noted. The quality of the reads was checked with FastQC v0.11.8 (12). Adapter sequences and low-quality regions in raw reads were removed with Trimmomatic v0.39 (13) using the following options: ILLUMINACLIP:TruSeq3-PE-2.fa:2:30:10; SLIDINGWINDOW:4:15; and MINLEN:55. Clean reads after trimming were assembled using SPAdes v3.14.1 (14) with the following options: --cov-cutoff, auto; and --careful. The obtained scaffolds were manually checked using Tablet v1.17.08.17 (15) to remove artifacts. Genome coverage was assessed with QUAST v5.1.0rc1 (16). The annotation was performed using the NCBI Prokaryotic Genome Annotation Pipeline v4.13 (17).

A total of 13,667,678 paired-end reads with a length of 76 bp were obtained from the sequencing. As a result, 13,154,612 clean reads with an average length of 75.6 bp were assembled into 131 scaffolds with 159-fold coverage. The scaffold *N*_50_ value is 92,182 bp, and the largest scaffold is 402,011 bp. The genome assembly size is 6,196,943 bp with an average G+C content of 66.9%. A total of 5,078 protein-coding genes (1,250 of which are coding hypothetical proteins), 50 tRNAs, 3 rRNAs, and 4 noncoding RNAs (ncRNAs) were predicted.

The genomic sequence of *L. capsici* VKM B-2533^T^ obtained in this work opens up new perspectives for discovering novel lytic agents and studying the mechanisms of their synthesis and regulation.

### Data availability.

The raw reads have been deposited in the NCBI SRA under the accession no. SRR12790239, and the whole-genome shotgun project has been deposited in DDBJ/ENA/GenBank under the accession no. JACXXL000000000. The version described in this paper is the first version, JACXXL010000000.
